# Recombination Hotspot/Coldspot Identification Combining Three Different Pseudocomponents via an Ensemble Learning Approach

**DOI:** 10.1155/2016/8527435

**Published:** 2016-08-25

**Authors:** Bingquan Liu, Yumeng Liu, Dong Huang

**Affiliations:** ^1^School of Computer Science and Technology, Harbin Institute of Technology, Harbin, Heilongjiang, China; ^2^School of Computer Science and Technology, Harbin Institute of Technology Shenzhen Graduate School, Shenzhen, Guangdong 518055, China; ^3^Key Laboratory of Network Oriented Intelligent Computation, Harbin Institute of Technology Shenzhen Graduate School, Shenzhen, Guangdong 518055, China

## Abstract

Recombination presents a nonuniform distribution across the genome. Genomic regions that present relatively higher frequencies of recombination are called hotspots while those with relatively lower frequencies of recombination are recombination coldspots. Therefore, the identification of hotspots/coldspots could provide useful information for the study of the mechanism of recombination. In this study, a new computational predictor called SVM-EL was proposed to identify hotspots/coldspots across the yeast genome. It combined Support Vector Machines (SVMs) and Ensemble Learning (EL) based on three features including basic kmer (Kmer), dinucleotide-based auto-cross covariance (DACC), and pseudo dinucleotide composition (PseDNC). These features are able to incorporate the nucleic acid composition and their order information into the predictor. The proposed SVM-EL achieves an accuracy of 82.89% on a widely used benchmark dataset, which outperforms some related methods.

## 1. Introduction

Meiotic recombination describes the process of alleles' exchange between homologous chromosomes during meiosis [1]. It can provide material for natural selection by producing diverse gametes. It might also contribute to the evolution of the genome via gene conversion or mutagenesis [2–4].

Although the exact location where recombination happens in the genome and the mechanism of recombination are still unclear, it has been assured that recombination plays an important role in promoting genome evolution. Therefore, several studies have been performed on chromosomes [5–7] and found that recombination presents a nonuniform distribution across the genome. Genomic regions that present relatively higher frequencies of recombination are called hotspots while those with relatively lower frequencies of recombination are called recombination coldspots [8, 9]. With the number of the sequenced genomes showing explosive growth, more reliable methods are urgently needed to be developed to identify the recombination spots.

The prediction of recombination hotspots or coldspots is still a challenging task, although much information can be acquired from the experiments. Recently, several computational models have been presented to identify the recombination hotspots/coldspots. For example, Liu et al. [10], based on sequence Kmer frequencies, proposed a model which combines the increment of diversity with quadratic discriminant analysis (IDQD). Later, this method was improved by adding gaps into the kmers [11]. Chen et al. presented a predictor called iRSpot-PseDNC trained with pseudo dinucleotide composition features [12].

The aforementioned methods extracted the features from DNA sequences in different aspects. For example, the model based on oligonucleotide frequencies considers the nucleic acid composition information. The iRSpot-PseDNC incorporates both the local nucleic acid composition information and the global information of the protein sequences. Therefore, it is reasonable to combine these complementary predictors to further improve the performance of recombination hotspot/coldspot identification. In this regard, three basic predictors trained with basic kmer (Kmer) [13], dinucleotide-based auto-cross covariance (DACC) [14, 15], and pseudo dinucleotide composition (PseDNC) [16], respectively, were combined via the framework of ensemble learning approach, and a novel predictor called SVM-EL was proposed. All these features can be easily generated by a recently proposed tool called Pse-in-One [17], which is able to generate various features only based on the DNA, RNA, or protein sequence information.

## 2. Materials and Methods

### 2.1. Benchmark Dataset

The benchmark datasets **S** was obtained from Liu et al. [10]:

(1) $$\begin{array}{c} S=S^{+}\cup S^{-}, \end{array}$$

where the subset **S** ^+^ contains 490 recombination hotspots, the subset **S** ^−^ contains 591 recombination coldspots, and the symbol ∪ represents the “union” in the set theory.

### 2.2. Feature Vectors Generated by Pse-in-One

SVM-EL is developed by combining the outcomes of three individual predictors which were trained by different features, including basic kmer (Kmer) [13], dinucleotide-based auto-cross covariance (DACC) [14, 15], and pseudo dinucleotide composition (PseDNC). These basic features can be generated by using Pse-in-One [17] which provides two approaches to generate feature vectors. One way is through the web server (http://bioinformatics.hitsz.edu.cn/Pse-in-One/) and another way is through the stand-alone tool (http://bioinformatics.hitsz.edu.cn/Pse-in-One/download/).

Suppose a DNA sequence **D** is

(2) $$\begin{array}{c} D=R_{1}R_{2}R_{3}R_{4}R_{5}\cdots R_{L}, \end{array}$$

where *L* represents the DNA sequence length and *R*_*i*_  (*i* = 1,2 ⋯ *L*) is the nucleic acid at the position *i*. Therefore, three basic features used in the current study can be described as follows.

#### 2.2.1. Kmer

Kmer [13] is an approach representing DNA sequences by the occurrence frequencies of kmers. The Kmer contains the local sequence-order information and it can be generated with the help of Pse-in-One by the following steps.

**Figure 1:**
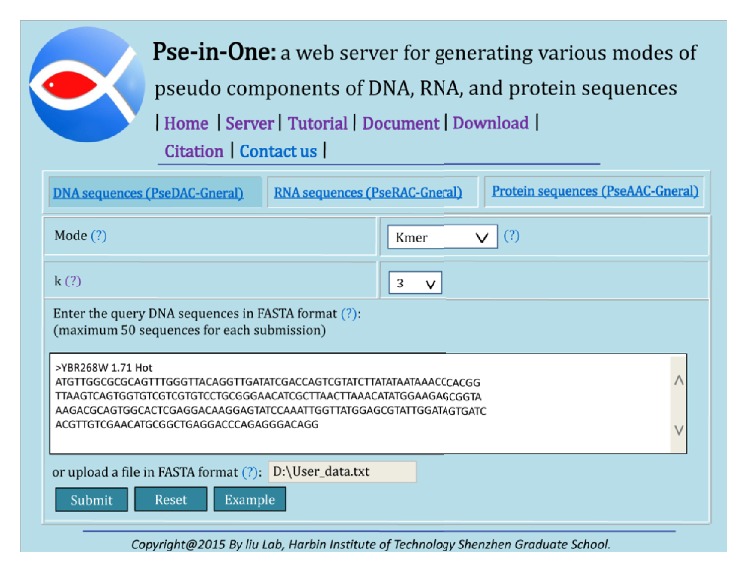
An example of the kmer features' generation by using Pse-in-One.

For web server approach, firstly, choose DNA sequences (PseDAC-General), then select Kmer in the tab of Mode, and set the value of *k*. Secondly, input or upload the DNA sequence file in FASTA format, click the Submit button, and then you will see the results and you can download them as a text file (Figure 1).

For stand-alone approach, Kmer features can be easily generated by using the following command line:

 ‘./kmer.py −f svm −l +1 3 DNA'

where −f svm represents the format of the output file which is the LIBSVM training data format, −l +1 represents the input file that contains positive samples only, *k* equals 3, and the sequence type is DNA.

#### 2.2.2. Dinucleotide-Based Auto-Cross Covariance (DACC)

Dinucleotide-based auto-cross covariance (DACC) [14, 15] is the combination of DAC [14, 15, 19] and DCC [14, 15]. The DAC measures the correlation between two dinucleotides for one DNA property [17]. The DCC approach measures the correlation between two dinucleotides for two different properties [17].

Given a DNA sequence **D** represented as (2), the DAC feature can be calculated as [17]

(3) DACμ,lag=∑i=1L−lag−1PμRiRi+1−P−μPμRi+lagRi+lag+1−P−μL−lag−1,P−μ=∑j=1L−1PμRjRj+1L−1,

where *μ* is the dinucleotide property index; *L* is the length of DNA sequence; lag represents the distance between two dinucleotides; *P*_*μ*_(*R*_*i*_ *R*_*i*+1_) represents the value of dinucleotide *R*_*i*_ *R*_*i*+1_ at position *i* for the dinucleotide property index *μ*; ${\overset{-}{P}}_{\mu }$ represents the average value of *P*_*μ*_(*R*_*i*_ *R*_*i*+1_) for a DNA sequence.

Given a DNA sequence **D** represented as (2), the DCC feature can be calculated as [17]

(4) DCCμ1,μ2,lag=∑i=1L−lag−1Pμ1RiRi+1−P−μ1Pμ2Ri+lagRi+lag+1−P−μ2L−lag−1,P−μ=∑j=1L−1PμRjRj+1L−1,

where *μ*_1_ and *μ*_2_ are two different dinucleotide property indices; *L* is the DNA sequence length; lag is the distance between two dinucleotides; *P*_*μ*_1__(*R*_*i*_ *R*_*i*+1_)(*P*_*μ*_2__(*R*_*i*_ *R*_*i*+1_)) represents the value of dinucleotide *R*_*i*_ *R*_*i*+1_ at position *i* for the dinucleotide property index *μ*_1_(*μ*_2_); ${\overset{-}{P}}_{\mu_{1}}({\overset{-}{P}}_{\mu_{2}})$ represents the average value of *P*_*μ*_1__(*R*_*i*_ *R*_*i*+1_)(*P*_*μ*_2__(*R*_*i*_ *R*_*i*+1_)) for a DNA sequence.

The features of DACC contain global sequence-order information, and it can be generated via Pse-in-One [17] which includes two generation approaches. The generation steps of DACC feature can be described as follows.

**Figure 2:**
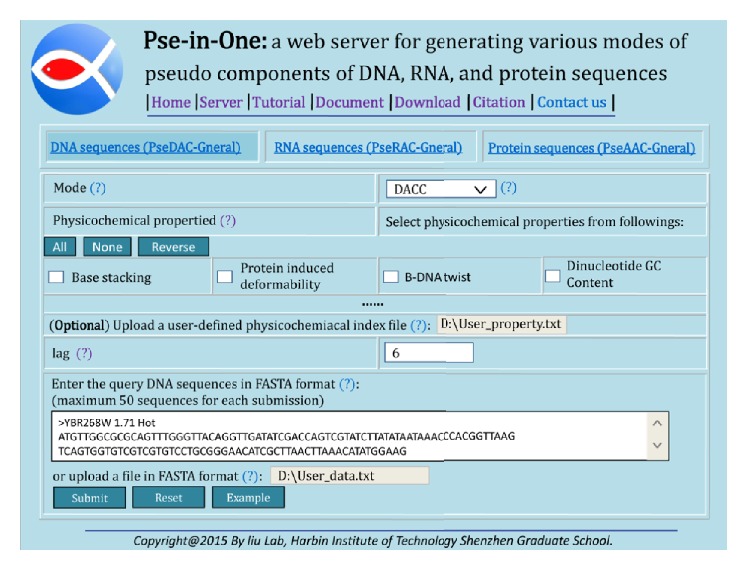
An example of the DACC features' generation by using Pse-in-One.

For web server approach, firstly, choose the DNA sequences (PseDAC-General) option, then select DACC in the tab of Mode, and set the value of lag. Secondly, upload a user-defined physicochemical index file called user_property and the values of fifteen dinucleotide physicochemical properties are shown in Table 1. Finally, input or upload the DNA sequence file in FASTA format, click the Submit button, and then you will see the results and you can download them as a text file (Figure 2).

For stand-alone approach, DACC features can be easily generated by using the following command line:

 ‘./acc.py −e user_property −f svm −l +1 3 DNA DACC'

where −e user_property represents the user-defined physicochemical index file, −f svm and −l +1 have the same meaning with the above command line, the parameter lag equals 3, the sequence type is DNA, and the method used is DACC.

#### 2.2.3. Pseudo Dinucleotide Composition (PseDNC)

Given a DNA sequence **D** represented as (2), the PseDNC feature vector **D** can be defined as [17]

(5) $$\begin{array}{c} D={\left[\;\begin{array}{cccccccc} d_{1} & d_{2} & d_{3} & \cdots & d_{16} & d_{16+1} & \cdots & d_{16+\lambda } \end{array}\;\right]}^{T}, \end{array}$$

where

(6) $$\begin{array}{c} d_{k}=\left\{\begin{array}{cc} \frac{f_{k}}{\sum_{i=1}^{16}f_{i}+w\sum_{j=1}^{\lambda }\theta_{j}}, & \left(1\leq k\leq 16\right),\\ \frac{w\theta_{k-16}}{\sum_{i=1}^{16}f_{i}+w\sum_{j=1}^{\lambda }\theta_{j}}, & \left(17\leq k\leq 16+\lambda \right), \end{array}\right. \end{array}$$

where *f*_*k*_  (1 ≤ *k* ≤ 16) represents the normalized frequency of dinucleotides along the DNA sequence; *w* (0 ≤ *w* ≤ 1) represents the weight factor; *λ* is the top counted tiers of the correlation in a DNA, *θ*_*j*_  (1 ≤ *j* ≤ *λ*) measures the correlation between dinucleotides in the DNA, which is defined as

(7) $$\begin{array}{c} \theta_{1}=\frac{1}{L-2}\overset{L-2}{\underset{i=1}{\sum }}\Theta \left(\;R_{i}R_{i+1},R_{i+1}R_{i+2}\;\right),\\ \theta_{2}=\frac{1}{L-3}\overset{L-3}{\underset{i=1}{\sum }}\Theta \left(\;R_{i}R_{i+1},R_{i+2}R_{i+3}\;\right),\\ \theta_{3}=\frac{1}{L-4}\overset{L-4}{\underset{i=1}{\sum }}\Theta \left(\;R_{i}R_{i+1},R_{i+3}R_{i+4}\;\right),\\ \vdots \\ \theta_{\lambda }=\frac{1}{L-1-\lambda }\overset{L-1-\lambda }{\underset{i=1}{\sum }}\Theta \left(\;R_{i}R_{i+1},R_{i+\lambda }R_{i+\lambda +1}\;\right),\\ \;\left(\lambda <L\right), \end{array}$$

where

(8) ΘRiRi+1,RjRj+1=1μ∑μ=1μPμRiRi+1−PμRjRj+12,

where *μ* represents the indices of the dinucleotide property; *P*_*μ*_(*R*_*i*_ *R*_*i*+1_)(*P*_*μ*_(*R*_*j*_ *R*_*j*+1_)) represents the value of dinucleotide *R*_*i*_ *R*_*i*+1_(*R*_*j*_ *R*_*j*+1_) at position *i*(*j*) for the dinucleotide property index *μ*.

Pseudo dinucleotide composition (PseDNC) [17] not only incorporates the local nucleic acid composition information and the global or long range information along the DNA sequences, but also incorporates the dinucleotide properties into feature vectors.

**Figure 3:**
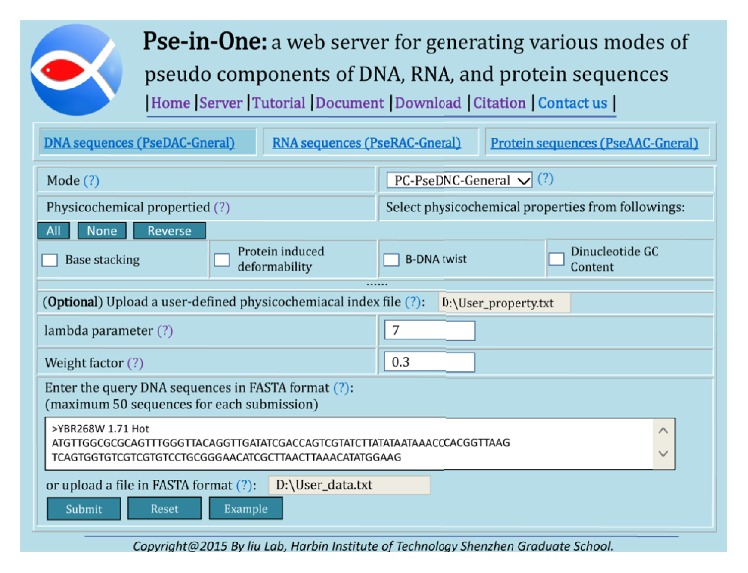
An example of the PseDNC features' generation by using Pse-in-One.

For web server approach, the generation steps of the feature vectors are similar to those of the DACC's. For web server approach, an example is shown in Figure 3.

For stand-alone approach, the command line is

 ‘./pse.py −e user_property −f svm −l +1 7 0.3 DNA PseDNC'

where −e user_property, −f svm, and −l +1 have the same meaning with the above command line, lambda equals 7, the value of weight equals 0.3, the sequence type is DNA, and the method used is PseDNC.

The meanings of all the parameters for these scripts are described in [17].

### 2.3. Support Vector Machine (SVM)

Support Vector Machine (SVM) is a kind of algorithm based on statistical learning theory proposed by Vapnik [20–22], which has been widely used for many bioinformatics tasks [23–27].

In the current study, the LIBSVM package version 3.21 [18] has been employed. The SVM parameters, the kernel width parameter *γ* and the regularization parameter *C*, were optimized via the grid tool provided by LIBSVM [18].

In the current study, three basic predictors are proposed, including SVM-Kmer, SVM-DACC, and SVM-PseDNC. The values of SVM-Kmer's parameters are shown as follows:

(9) C=27,γ=2,k=6.

The values of SVM-DACC's parameters are shown as follows:

(10) C=23,γ=2−3,lag=6.

The values of SVM-PseDNC's parameters are shown as follows:

(11) C=213,γ=23,λ=7,w=0.3.

### 2.4. Ensemble Learning

**Figure 4:**
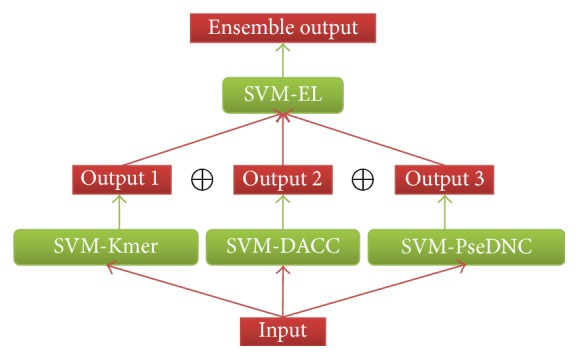
The basic framework for an ensemble classifier.

In machine learning, ensemble learning is the process by which multiple classifiers are constructed and combined based on the same dataset to obtain a better performance than a single classifier [28, 29] and existing popular multiobjective optimization evolutionary algorithms can be used for ensemble learning [30, 31]. Ensemble classifier also performed well in several bioinformatics problems. In the current study, the basic framework for an ensemble classifier is illustrated in Figure 4. The final results are obtained by fusing three individual classifier outcomes, as illustrated below.

Suppose the ensemble classifier *ℂ* is defined as

(12) $$\begin{array}{c} C=C_{1}⊕C_{2}⊕C_{3}, \end{array}$$

where *ℂ*_1_ represents the classifier SVM-Kmer, *ℂ*_2_ represents the classifier SVM-DACC, and *ℂ*_3_ represents the classifier SVM-PseDNC. The symbol ⊕ denotes the fusing operator.

Therefore, the process of the ensemble classifier can be formulated as follows:

(13) $$\begin{array}{c} R_{j}=\frac{1}{3}P_{i}\left(\;S,L_{j}\;\right),\;\left(i=1,2,3;\;\;j=1,2\right), \end{array}$$

where *L*_1_ is the set only containing recombination hotspots and *L*_2_ is the set of recombination coldspots. *P*_*i*_(**S**, *L*_*j*_) is the probability for DNA sequence **S** which belongs to category *L*_*i*_ obtained by the *i*th basic classifier.

Thus, which category the query DNA **S** belongs to is to be determined by using its average probability calculated by (13); that is, suppose that

(14) $$\begin{array}{c} R_{\mu }=\max\left\{\;R_{1},R_{2}\;\right\}, \end{array}$$

where the operator max represents selecting a lager value in the brackets, and the subscript *μ* represents the query DNA **S** belonging to category *L*_*μ*_.

### 2.5. Criteria for Performance Evaluation

The prediction results can be divided into true positive (TP), false negative (FN), false positive (FP), and true negative (TN) [32]. In the current study, jackknife test [33–37] was employed and four kinds of evaluation indexes were adopted, including Sensitivity (Se), Specificity (Sp), Accuracy (Acc), and Matthew's Correlation Coefficient (Mcc). They are described as

(15) Se=TPTP+FN×100%,Sp=TNTN+FP×100%,Acc=TP+TNTP+TN+FP+FN×100%,Mcc=TP×TN−FP×FNTP+FPTP+FNTN+FPTN+FN.

## 3. Results and Discussion

### 3.1. Performance of the Three Basic Classifiers

As an inherent property, sequence-order is important for the classification of DNA sequences. So, three basic methods based on sequence-order information are adopted to identify recombination hotspots/coldspots.  Table 2 shows the performance of the three methods. According to the table, we can see that SVM-DACC and SVM-PseDNC outperform SVM-Kmer on the prediction accuracy index. The main reason is that SVM-Kmer is only based on local sequence-order information, while both of SVM-DACC and SVM-PseDNC also contain global sequence-order information.

### 3.2. The Performance of the Three Basic Predictors Can Be Further Improved by Using Ensemble Learning

**Figure 5:**
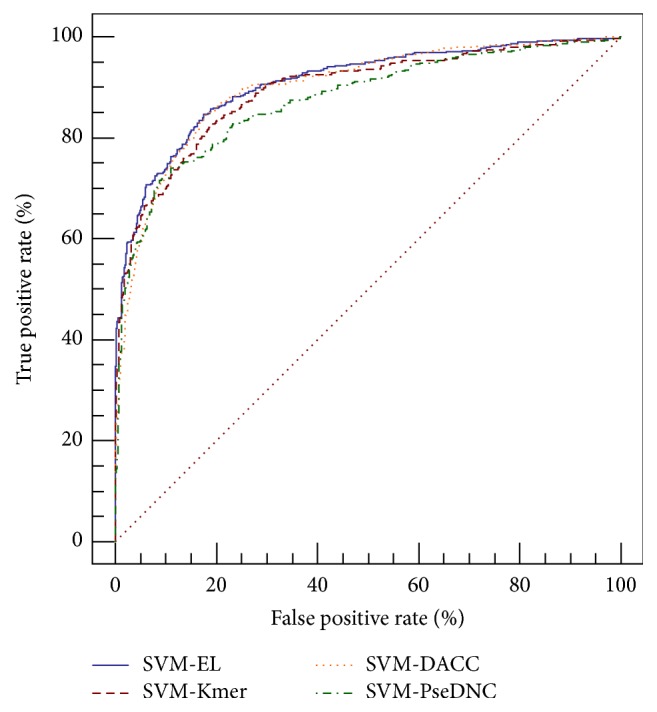
The comparison of different predictors for hotspots/coldspots identification. The areas under ROC curves (AUC) of SVM-EL, SVM-DACC, SVM-Kmer, and SVM-PseDNC are 0.91, 0.90, 0.89, and 0.87, respectively.

Based on the analysis above, we have proposed three basic predictors for identifying recombination hotspots/coldspots. These methods capture DNA information from different aspects. Therefore, we presented a complementary method SVM-EL which can fuse these basic methods to improve the prediction performance. The performance of SVM-EL is shown in Table 2, from which we can see that SVM-EL outperforms the three basic methods. Besides, the corresponding receiver operating characteristic (ROC) curves of the four classifiers were drawn in Figure 5. AUC, the area under the ROC curve, is often used to indicate the performance of a classifier: the larger the value, the better the classifier.

As shown in Figure 5, the predictor SVM-EL showed the top performance, outperforming three basic methods: SVM-Kmer, SVM-DACC, and SVM-PseDNC.

### 3.3. Comparison with Other Related Predictors

Two state-of-the-art methods, IDQD [10] and iRSpot-PseDNC, were selected to compare with the proposed SVM-EL.  Table 3 shows the results of various methods on the benchmark dataset.

According to Table 3, we can see that SVM-EL outperforms the other methods. The main reason is that IDQD and SVM-Kmer only consider local sequence-order information, and iRSpot-PseDNC, SVM-DACC, and SVM-PseDNC improved them by incorporating global sequence-order information. However, SVM-EL not only incorporates the local nucleic acid information, but also incorporates the global information. Therefore, we conclude that SVM-EL would be a useful tool for hotspots/coldspots identification.

## 4. Conclusion

In this article, we proposed a predictor called SVM-EL for yeast hotspot/coldspot identification, which combines Support Vector Machine (SVM) with Ensemble Learning (EL). The approach combined with different predictors trained by different features contributes to the improvement of prediction accuracy. SVM-EL is trained by different features, including basic kmer (Kmer), dinucleotide-based auto-cross covariance (DACC), and pseudo dinucleotide composition (PseDNC). All these features can be generated by Pse-in-One [17], which is a powerful web server for generating various DNA, RNA, or protein features. It also provides a stand-alone version to users, which is easy to use. Via jackknife test, it was observed that the predictor outperforms other predictors. In the future, we will consider using other approaches for yeast hotspot/coldspot identification, such as bioinspired computing models [38–45].

## Figures and Tables

**Table 1:** The values of fifteen DNA dinucleotide properties.

	AA/TT	AC/GT	AG/CT	AT	CA/TG	CC/GG	CG	GA/TC	GC	TA
F-roll	0.04	0.06	0.04	0.05	0.04	0.04	0.04	0.05	0.05	0.03
F-tilt	0.08	0.07	0.06	0.10	0.06	0.06	0.06	0.07	0.07	0.07
F-twist	0.07	0.06	0.05	0.07	0.05	0.06	0.05	0.06	0.06	0.05
F-slide	6.69	6.80	3.47	9.61	2.00	2.99	2.71	4.27	4.21	1.85
F-shift	6.24	2.91	2.80	4.66	2.88	2.67	3.02	3.58	2.66	4.11
F-rise	21.34	21.98	17.48	24.79	14.51	14.25	14.66	18.41	17.31	14.24
Roll	1.05	2.01	3.60	0.61	5.60	4.68	6.02	2.44	1.70	3.50
Tilt	−1.26	0.33	−1.66	0.00	0.14	−0.77	0.00	1.44	0.00	0.00
Twist	35.02	31.53	32.29	30.72	35.43	33.54	33.67	35.67	34.07	36.94
Slide	−0.18	−0.59	−0.22	−0.68	0.48	−0.17	0.44	−0.05	−0.19	0.04
Shift	0.01	−0.02	−0.02	0.00	0.01	0.03	0.00	−0.01	0.00	0.00
Rise	3.25	3.24	3.32	3.21	3.37	3.36	3.29	3.30	3.27	3.39
Energy	−1.00	−1.44	−1.28	−0.88	−1.45	−1.84	−2.17	−1.30	−2.24	−0.58
Enthalpy	−7.60	−8.40	−7.80	−7.20	−8.50	−8.00	−10.60	−8.20	−9.80	−7.20
Entropy	−21.30	−22.40	−21.00	−20.40	−22.70	−19.90	−27.20	−22.20	−24.40	−21.30

**Table 2:** Results on benchmark dataset for different predictors proposed in the current study.

Predictor	Test method	Se (%)	Sp (%)	Acc (%)	MCC
SVM-Kmer^a^	Jackknife	75.92	86.29	81.59	0.628
SVM-DACC^b^	Jackknife	76.12	87.99	82.61	0.649
SVM-PseDNC^c^	Jackknife	72.04	90.69	82.24	0.644
SVM-EL	Jackknife	76.33	88.33	82.89	0.654

^a^The parameters used are *k* = 6 for SVM-Kmer and *C* = 2^7^ and *γ* = 2 for LIBSVM [18].

^b^The parameters used are lag = 6 for SVM-DACC and *C* = 2^3^ and *γ* = 2^−3^ for LIBSVM [18].

^c^The parameters used are *λ* = 7 and *w* = 0.3 for SVM-PseDNC and *C* = 2^13^ and *γ* = 2^3^ for LIBSVM [18].

**Table 3:** Results on benchmark dataset for different predictors.

Predictor	Test method	Se (%)	Sp (%)	Acc (%)	MCC
IDQD^a^	5-fold	79.40	81.00	80.30	0.603
iRSpot-PseDNC^b^	Jackknife	73.06	89.49	82.04	0.638
SVM-EL	Jackknife	76.33	88.33	82.89	0.654

^a^From Liu et al. [10].

^b^From Chen et al. [12].
